# TACH101, a first-in-class pan-inhibitor of KDM4 histone demethylase

**DOI:** 10.1097/CAD.0000000000001514

**Published:** 2023-03-24

**Authors:** Chandtip Chandhasin, Van Dang, Frank Perabo, Joselyn Del Rosario, Young K. Chen, Ellen Filvaroff, Jeffrey A. Stafford, Michael Clarke

**Affiliations:** a Tachyon Therapeutics Inc.; b Bristol Myers Squibb; c 858 Therapeutics; d Stanford University, Stanford, California, USA

**Keywords:** epigenetic, histone lysine demethylase, KDM4, TACH101

## Abstract

Histone lysine demethylase 4 (KDM4) is an epigenetic regulator that facilitates the transition between transcriptionally silent and active chromatin states by catalyzing the removal of methyl groups on histones H3K9, H3K36, and H1.4K26. KDM4 overamplification or dysregulation has been reported in various cancers and has been shown to drive key processes linked to tumorigenesis, such as replicative immortality, evasion of apoptosis, metastasis, DNA repair deficiency, and genomic instability. KDM4 also plays a role in epigenetic regulation of cancer stem cell renewal and has been linked to more aggressive disease and poorer clinical outcomes. The KDM4 family is composed of four main isoforms (KDM4A-D) that demonstrate functional redundancy and cross-activity; thus, selective inhibition of one isoform appears to be ineffective and pan-inhibition targeting multiple KDM4 isoforms is required. Here, we describe TACH101, a novel, small-molecule pan-inhibitor of KDM4 that selectively targets KDM4A-D with no effect on other KDM families. TACH101 demonstrated potent antiproliferative activity in cancer cell lines and organoid models derived from various histologies, including colorectal, esophageal, gastric, breast, pancreatic, and hematological malignancies. *In vivo*, potent inhibition of KDM4 led to efficient tumor growth inhibition and regression in several xenograft models. A reduction in the population of tumor-initiating cells was observed following TACH101 treatment. Overall, these observations demonstrate the broad applicability of TACH101 as a potential anticancer agent and support its advancement into clinical trials.

## Introduction

Histone modification, in which specific lysine residues undergo acetylation, methylation, demethylation, ubiquitination, and other modifications, is an important process in epigenetic regulation during embryonic development, gene transcription, cell cycle progression, and DNA replication and repair. In this report, we describe a novel histone lysine demethylase 4 (KDM4) inhibitor with high selectivity for KDM4 family members A–D and potent anticancer activity. TACH101 is a synthetic small molecule that demonstrated antiproliferative activity across a large panel of cancer cell lines, apoptotic inducibility, as well as effective tumor growth inhibition in multiple xenograft cancer models.

KDM4 enzymes belong to the Jumonji (JMJ) C family comprised of four main isoforms: KDM4A, KDM4B, KDM4C, and KDM4D. KDM4A−C are structurally similar, containing two JMJ domains N and C (which house the catalytic activity) followed by noncatalytic domains comprised of two plant homeodomains and two Tudor domains that determine substrate specificity and control enzyme activity. KDM4D is a truncated protein containing only two JMJ domains. The substrates for KDM4 are histone H3K9 and histone H3K36 in the form of di- or trimethylation (H3K9me2/3, H3K36me2/3) [1–5], and the linker di- or trimethyl histone H1.4 K26 (H1.4K26me2/3) [6]. KDM4 stimulates or represses the expression of specific target genes by demethylating these histones.

Not surprisingly, there is substantial functional redundancy among the KDM4 isoforms. Individual genetic deletions of KDM4A, KDM4B, or KDM4C in mice did not significantly impair cellular proliferation, embryonic stem cell self-renewal, or embryonic development [7]. However, double knockout of KDM4A/4C or triple knockout of KDM4A/4B/4C resulted in significant reductions in cell proliferation and/or embryonic lethality [7,8]. Thus, inhibition of one isoform appears to be ineffective; instead, pan-inhibition targeting multiple KDM4 isoforms is required.

Overexpression or deregulation of KDM4 can lead to aberrant gene activation triggering numerous tumorigenic pathways, which has been described in several tumor types including breast, colorectal, brain, renal, pancreatic, gastric, lung, testicular, prostate, bladder, melanoma, squamous cell, and lymphoma [1,4,9–17]. Conversely, knockdown studies of KDM4 showed antiproliferative effects in cancers, including AML [8], lymphoma [18], breast [12,13,19–21], esophageal [1], lung [22], colon [15,23–32], gastric [29,31,33,34], prostate [35–37], and ovarian cancers [38]. Interestingly, knockdown of *KDM4B* in colorectal cancer cells stimulates the DNA damage response, which subsequently induces cell cycle arrest, apoptosis, and senescence [24]. KDM4 has also been shown to be necessary for the self-renewal of embryonic stem cells and induction of pluripotent stem cell generation [39–42]. In addition, KDM4 serves as an epigenetic control for the stem-like properties of colorectal cancer [43].

TACH101 is a novel, small-molecule pan-inhibitor of KDM4 that selectively targets isoforms A–D with no effect on other KDM families. TACH101 demonstrated potent antiproliferative activity in cancer cell lines and organoid models derived from various histologies, including colorectal, esophageal, gastric, breast, pancreatic, and hematological malignancies. TACH101 treatment led to efficient tumor growth inhibition in several xenograft models *in vivo*. The broad spectrum of TACH101 therapeutic applicability shows tremendous promise against both hematologic and solid tumors, and to date, is the first compound of this class to enter clinical trials.

## Materials and methods

### Mechanism of inhibition studies

TACH101 inhibition of KDM4C demethylation of H3K9me3 to the dimethyl peptide H3K9me2 was measured at various TACH101 concentrations in the presence of alpha-ketoglutarate (α-KG) at multiple concentrations using the time-resolved fluorescence resonance energy transfer (TR-FRET) LANCE detection system. KDM4C was prepared at a concentration of 2.4 μg/μl (>82% purity) in 40 mmol/l tris (pH 8.0), 110 mmol/l sodium chloride, 2. 2 mmol/l potassium chloride, 16 mmol/l glutathione, and 20% glycerol. The assay was performed in a 384-well multiwell plate format under the following reaction conditions shown as final concentrations: 4 nmol/l KDM4C, 300 nmol/l H3K9me3 biotin-labeled peptide (Anaspec Cat #: 64360) and various concentration of α-KG ranged from 1 to 128 µmol/l in an assay buffer of 50 mmol/l HEPES (pH 7.3), 0.5 mmol/l tris (2-carboxyethyl) phosphine, 0.005% (w/v) Brij-35, 0.02% (w/v) BSA, 5 µmol/l ferrous (II) sulfate, and 50 µmol/l ascorbic acid. Each test well also contained 1% DMSO as a negative control, an inhibitor at ≥100 × IC_50_ concentration in 1% DMSO as a positive control, or serially diluted TACH101 at 0.4–200 µmol/l in 1% DMSO. The reaction mixtures were incubated at ambient temperature for 10 min and terminated in each test well by adding 6 μl of a solution containing 5 mmol/l EDTA, 1× LANCE detection buffer, 100 nmol/l PhycoPro GT5 APC-Streptavidin, and 2 nmol/l LANCE Eu-H3K9me2 Ab (PerkinElmer Cat#: TRF0403). The final demethylated H3K9me2 reaction product for each test well was determined quantitatively using an EnVision Multilabel Reader (PerkinElmer, Waltham, Massachusetts, USA) in TR-FRET detection mode (excitation at 320 nm, emission at 615 nm, and 665 nm). The ratio was calculated (665/615) for each well and was fitted to determine the inhibition constant (IC_50_).

Time-dependent inhibition of H3K9me3 demethylation was measured at various TACH101 concentrations preincubated with KDM4C for 2.5–90 min. Assays were performed in multiwell plates under the following reaction conditions shown as final concentrations: 300 nmol/l H3K9me3 biotin-labeled peptide, 100 µmol/l α-KG, serially diluted TACH101, and 3 nmol/l KDM4C. The reaction mixtures were incubated for 10 min, after which the reactions were terminated and quantitatively assessed by LANCE TR-FRET detection. Curves were fitted using nonlinear regression, and the IC_50_ value for each preincubation time was computed as the concentration at which inhibition was half-maximal. The IC_50_ values were plotted against the preincubation time, and a nonlinear regression curve was fitted.

### Lysine demethylase (KDM) isoform selectivity of TACH101

The ability and selectivity of TACH101 to inhibit the activity of KDM family members were evaluated using biotin-labeled methylated histone H3 peptide as a substrate. Assays were performed in a 384-well plate based on the conditions described in Supplementary Table S1, Supplemental Digital Content 1, http://links.lww.com/ACD/A498. All biotinylated H3 peptides were purchased from Anaspec. The demethylated product was quantitatively assessed using either LANCE TR-FRET detection, by the addition of the europium-labeled antibody (Perkin Elmer) and Phycolink streptavidin-allophycocyanin (Prozyme), or, for AlphaScreen assays, by the addition of the specified antibody followed by the addition of streptavidin-coated donor beads and protein A-conjugated acceptor beads (Perkin Elmer). Plates were read by an Envision Multilabel Reader in either TR-FRET or AlphaScreen mode. Curves were fitted using nonlinear regression, and IC_50_ values were computed as the concentrations at which inhibition was half-maximal.

### Cellular histone demethylation assay

KYSE-150 cells stably overexpressing KDM4C (KYSE-150^+KDM4C^) (Lenti construct from Origene Cat#: CW102419) were treated with or without TACH101, and endogenous trimethyl histone H3 lysine 36 (H3K36me3) levels were quantitatively assessed. Homogeneous time-resolved fluorescence (HTRF)-based H3K36me3 assays (Cisbio, Bedford, Massachusetts, USA) were performed in multiwell plates seeded with KYSE-150^+KDM4C^ cells. Assays were initiated by the addition of a negative control (DMSO), positive control (potent internal KDM4C inhibitor QC5843 at ≥100 × IC_50_ concentration), or serially diluted TACH101. The cultures were incubated for 24 h, after which the reactions were terminated by cell lysis. Endogenous H3K36me3 was quantitatively assessed in a sandwich HTRF assay that used a europium cryptate-labeled anti-H3K36me3 donor (CST 4909) and a d2-labeled antihistone H3 acceptor. The final trimethylated H3K36me3 reaction product for each test well was determined quantitatively using an EnVision Multilabel Reader in the TR-FRET detection mode, and the readout for cells treated with TACH101 was expressed as a percentage of the positive control. The percentage of control values was plotted against the corresponding concentrations of TACH101.

Orthogonal immunoblot assays were performed in multiwell plates seeded with KYSE-150^+KDM4C^ cells. Assays were initiated by adding serially diluted TACH101. Cultures were incubated for 24 h, after which the reactions were terminated by cell lysis followed by SDS-polyacrylamide electrophoresis and immunoblotting for actin and H3K36me3 using monoclonal anti-β-actin antibody (Sigma-Aldrich Cat#: A2228) and trimethyl-histone H3 (Lys36) (D5A7) XP Rabbit mAb (Cell Signaling Technology Cat#: 4909, RRID:AB_1950412), respectively. Endogenous H3K36me3 was quantitatively assessed by plotting the ratio of H3K36me3/actin signals following densitometry versus the TACH101 concentration. The IC_50_ values were determined from nonlinear regression curves as the concentrations at which inhibition was half-maximal. The IC_50_ was expressed as the mean IC_50_ ± SD for ‘N’ assays.

### Cell proliferation assays

Jurkat (ATCC# TIB-152, RRID:CVCL_0367), MDA-MB-231 (ATCC# HTB-26), KYSE-150 (RRID:CVCL_1348), MM.1s (ATCC# CRL-2974), HL-60 (ATCC# CCL-240, RRID:CVCL_0002), HT-29 (ATCC# HTB-38, RRID:CVCL_0320), MCF-7 (ATCC# HTB-22, RRID:CVCL_0031), Loucy (ATCC# CRL-2629), and IMR-90 (ATCC# CCL-186, RRID:CVCL_0347) cell lines were seeded in 384-well plates and cultured for 24 h in a humidified incubator at 37 °C to promote adherence. TACH101 inhibition of cell proliferation was determined using Cell Proliferation ELISA, BrdU (colorimetric) assay (Roche/Fisher Cat #: 11647229001), Promega Cell Titer Glo 2.0 assay kit (Promega Cat #: G-9243), or Promega Cell AQueous MTS assay (Promega Cat #: G-5440). Assays were initiated in individual test wells by adding DMSO as a negative control, panobinostat as a positive control, or serially diluted TACH101. The cultures were incubated for 168 h, after which the number of viable cells in each test well was assessed using the methods described above, according to the manufacturer’s instructions. Readouts were performed using an EnVision Multilabel Reader (PerkinElmer, Waltham, Massachusetts, USA), and the results were expressed as a percentage of the negative control. The percentage of control values was plotted against the corresponding TACH101 concentration, and the relative IC_50_ value was determined from a nonlinear regression curve as the concentration at which inhibition was half-maximal.

In a larger screening study, TACH101 was profiled against a panel of 301 cell lines from various tissues of origin (OncoPanel; HD Biosciences) (Supplementary Table S2, Supplemental Digital Content 1, http://links.lww.com/ACD/A498). Each cell line was treated with TACH101 at 10 different doses (10–0.000 5µmol/l with 1:3 serial dilution) in a singlet, and the number of viable cells in the culture was determined using a CellTiter-Glo luminescent cell viability assay (Promega Cat#: G7571) based on quantification of the ATP present at the end of 7 days of exposure.

### Organoid cell viability assay methods

The following patient-derived cancer organoid models were originally sourced from Quanticel during the early development of the compound: colon cancer (T002C, SU60, SU62, SU34, SU103, T035C, SU106), pancreatic cancer (PA0165F, PA0143F, T016P, T028P), and breast cancer (FS53).

Colony formation and viability assays were performed in multiwell plates seeded with organoids using an in-vitro culture system composed of a matrigel layer, media containing a defined set of growth factors, and coculture with Wnt3A-secreting mouse embryonic fibroblasts. Assays were initiated by adding DMSO as a negative control, an inhibitor panobinostat at ≥100 × IC_50_ concentration as a positive control, or serially diluted TACH101. The cultures were incubated for 168 or 144 h, after which the number of viable organoids in each test well was assessed using one of two detection methods: the CellTiter-Glo luminescent cell viability assay or staining with calcein AM, followed by imaging and colony counting. The colony count or viability of cells treated with TACH101 is expressed as a percentage of the negative control. Aggregated percent of control values were plotted against the corresponding TACH101 concentration, and the IC_50_ value was determined from a nonlinear regression curve as the concentration at which inhibition was half-maximal.

### Apoptotic and cell cycle arrest assays

Apoptotic assays were performed using the KYSE-150, MDA-MB-231, HT-29, and HCC1937 (ATCC# CRL-2336, RRID:CVCL_0290) cell lines. Individual assays were initiated in separate test wells by adding either DMSO as a negative control, the inhibitor staurosporine at 500 nmol/l (≥100 × IC_50_ concentration) as a positive control, panobinostat (12.5 µmol/l) as a standard, or serially diluted TACH101. Duplicate cultures were incubated for 72 h, after which the reactions were terminated via cell lysis. Apoptosis was quantitatively assessed using luminescence detection of the cleavage product of a luminogenic Asp-Glu-Val-Asp-Gly (DEVD) tetrapeptide substrate selective for caspases −3 and −7. The readout was used to calculate the percent activity for each TACH101 concentration normalized to that of the panobinostat. Curves were fitted using nonlinear regression, and EC_50_ values were computed as the concentrations at which induction was half-maximal of activity induced by panobionstat.

Cell cycle assays were performed in multiwell culture plates seeded with MDA-MB-231 cells. Assays were initiated by the addition of a DMSO negative control, 1 µmol/l panobinostat, or TACH101 at 0.01 or 0.1 µmol/l. The cells were cultured for 48 or 72 h, after which they were harvested, washed, and resuspended in cold PBS. The cells were fixed by slowly adding nine volumes of 100% ethanol and then stored at ≤4 °C for ≥2 h. Fixed cells were washed in cold PBS, resuspended in propidium iodide (PI) staining solution [1× PBS with 50 µg/ml PI, 0.1 mg/ml RNAse A, 0.05% (v/v) Triton X-100], and incubated for 15 min at 37 °C before analysis by flow cytometry. Cell cycle analysis was performed using the MultiCycle AV DNA analysis software (Phoenix Flow Systems, San Diego, California, USA).

### Tumorigenicity assays

Female nonobese diabetic/severe combined immunodeficiency gamma (NSG) mice engrafted with SU60 cells (seven mice in each group) were administered TACH101 at 40 mg/kg orally (PO) once daily (QD) ×21 days. The day after the last dose, three tumors nearest to the mean size from each group were dissociated, and single cells were used to perform flow cytometry analysis of the tumorigenic cell population and functional in-vivo tumorigenicity assay (limiting dilution assay). For flow cytometry analysis, cells were labeled with markers CD44 and EpCAM, and the proportion of tumorigenic CD44^High^EpCAM^+^ cells in the cell population was quantified. For the limiting dilution assay, dissociated cells (50–1000) from treated and vehicle control groups were injected subcutaneously in a limiting dilution manner into NSG mice, and tumor volumes were measured.

### Mouse xenograft studies

All experiments were performed in accordance with the U.S. Code of Federal Regulations 9 CFR 2.31 (Institutional Animal Care and Use Committee, IACUC) and approved by the local IACUC and the ethical committee of Celgene Quanticel Research. The protocols are described in Supplementary Materials, Supplemental Digital Content 1, http://links.lww.com/ACD/A498, and the Methods section.

## Results

### TACH101 is an alpha-ketoglutarate competitive KDM4 inhibitor with slow off-rate kinetics

**Fig. 1:**
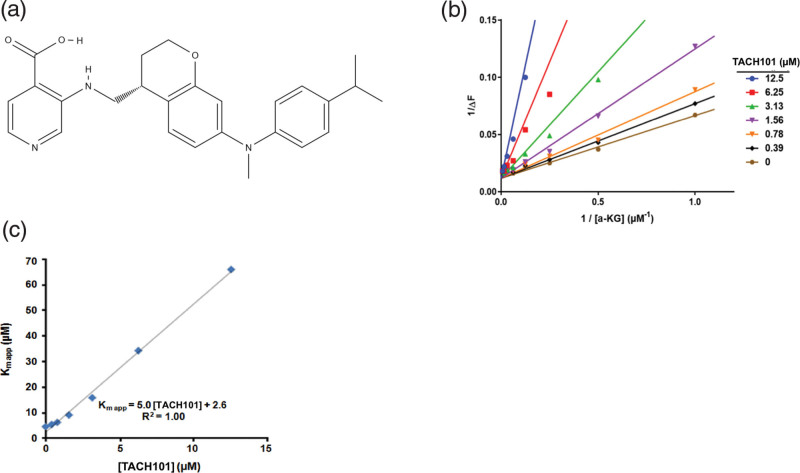
(a) TACH101 chemical structure. C_26_H_29_N_3_O_3_ • C_6_H_14_N_2_O_2_; MW 577.73 g/mol (lysine salt). (b) Lineweaver-Burk Plot of TACH101 inhibition of KDM4C. Inhibition of demethylation of H3K9me3 was measured for various (TACH101) and (α-KG). Plots of 1/V0 versus 1/(α-KG) for each (TACH101) exhibited the same y-intercept for inhibited and uninhibited KDM4C with slopes proportional to the (TACH101) establishing the mechanism as competitive inhibition. (c) Plot of α-Ketoglutarate Apparent Michaelis-Menton Constant versus (TACH101). The α-KG Km app for each (TACH101) was determined as slope/y-intercept quotient from the corresponding Lineweaver-Burk plot. The resulting Km app values were plotted versus the corresponding (TACH101) and the line-of-best-fit was determined. KDM4, histone lysine demethylase 4; MW, molecular weight; α-KG, alpha-ketoglutarate.

The chemical structure of TACH101 is shown in Fig. 1a. TACH101 was shown to exhibit its inhibitory mechanism by competing with KDM4’s catalytic cofactor, α-KG with an apparent inhibition constant (Ki) of 0.52 µmol/l for the initial enzyme-inhibitor complex (Fig. 1b and c). Time-dependent inhibition of KDM4C by TACH101 was also investigated. The k_on_ ± SD was calculated as 0.27 ± 0.01 μmol/l^−1^ min^−1^ and the k_off_ constant and half-life for KDM4C·TACH101* were calculated as 0.010 ± 0.001 min^−1^ and 68 ± 4 min, respectively. The binding of TACH101 to KDM4C was reversible, but the slow off-rate indicated extended engagement of TACH101 to the target. Ki* was calculated from the K_off_/K_on_ rate constant at 0.038 ± 0.004 µmol/l. These results are consistent with a two-step slow binding mechanism, in which fast initial binding is followed by slow isomerization to a KDM4C·TACH101* steady state. The transition from KDM4C·TACH101 to KDM4C·TACH101* decreases the inhibition constant 14-fold from 0.52 to 0.038 µmol/l at 300 nmol/l H3K9me3.

### TACH101 is a highly selective KDM4 inhibitor

The selectivity of TACH101 was assessed against KDM family members 2–7. TACH101 was observed to exhibit selective and potent inhibition of KDM4 isoforms A–D (IC_50_ ≤ 0.080 µmol/l) when preincubated for 60 min before the initiation of inhibition assays. Without preincubation, TACH101 was less potent in inhibiting KDM4 isoforms A and C than the corresponding outcomes with incubation (Supplementary Table S3, Supplemental Digital Content 1, http://links.lww.com/ACD/A498). Without preincubation, TACH101 inhibited KDM5 family members at potencies ranging from 0.14 to 0.40 µmol/l. However, binding was not time-dependent, and TACH101 IC_50_ following preincubation was slightly less potent for KDM5B and significantly less potent for KDM5A, relative to no preincubation. TACH101 showed significantly lower potency against KDM2, KDM3, KDM6, and KDM7 family members with or without preincubation.

### TACH101 inhibits histone demethylation in cells

**Fig. 2:**
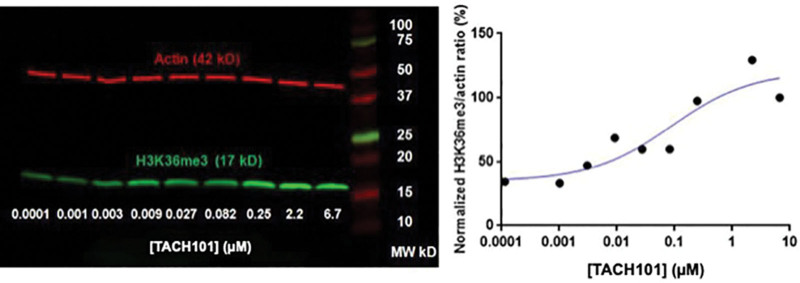
TACH101 inhibition of H3K36me3 demethylation in KYSE-150^+KDM4C^ cells in a representative orthogonal immunoblot assay. Left panel = endogenous H3K36me3 was quantitatively assessed by SDS-polyacrylamide electrophoresis and immunoblotting for actin and H3K36me3. Reference bands (Dual Xtra Standards, Bio-Rad, Hercules, California, USA) are shown on the left labeled with their molecular weight (MW) in kiloDaltons (kD). The immunoblot shows change in staining intensity for actin (red bands, MW = 42 kD) and H3K36me3 (green bands, MW = 17 kD) in response to TACH101 at various concentrations. Right panel = normalized ratio of band intensities for H3K36me3 and actin versus concentration of TACH101 (µmol/l) was plotted. H3K36me3, trimethyl histone H3 lysine 36; KDM4, histone lysine demethylase 4.

To assess the cellular on-target engagement of TACH101, demethylation of endogenous cellular H3K36me3 in the presence or absence of TACH101 was examined. As shown in Table 1, TACH101 potently inhibited the demethylation of endogenous H3K36me3, yielding a mean IC_50_ ± SD value of 0.0004 ± 0.0003 µmol/l in an HTRF-based assay and a mean IC_50_ ± SD value of 0.085 ± 0.004 in an immunoblot assay. A representative titration curve for data aggregated across the three HTRF H3K36me3 assays was constructed and is presented in Supplementary Fig. S1, Supplemental Digital Content 1, http://links.lww.com/ACD/A498. In these assays, the maximum inhibition by TACH101, relative to the positive control, was ~40%. Inhibition of demethylation was confirmed by two orthogonal immunoblotting assays, which yielded a mean IC_50_ ± SD value of 0.085 ± 0.004 µmol/l (Fig. 2).

**Table 1:** TACH101 half-maximal inhibition value for H3K36me3 demethylation in KYSE-150^+KDM4C^ cells

Assay	Mean IC_50_ (µmol/l)	SD (µmol/l)	*N (n*)
HTRF	0.0004	0.0003	4 (3)
Immunoblot	0.085	0.004	2 (1)

H3K36me3, trimethyl histone H3 lysine 36; HTRF, homogeneous time-resolved fluorescence; IC_50_, relative half-maximal inhibitory concentration (µmol/l); *N (n*), ‘*N*’ number of experiments and ‘*n*’ different TACH101 batches tested.

### TACH101 inhibits proliferation of cancer cell lines and organoid models

The IC_50_ values for TACH101 inhibition of cell proliferation were determined for various cancer cell lines (representing both solid and hematological cancers) and one normal human fibroblast cell line. Except for the normal human fibroblast line (IMR-90; IC_50_ > 1.0 µmol/l), TACH101 treatment for 168 h demonstrated potent antiproliferative activity across the panel (IC_50_ values ranged from 0.0027 to 0.037 µmol/l) (Table 2).

**Table 2:** TACH101 half-maximal inhibition values for various cancer cell lines

Cell line	Description	IC_50_ (µmol/l)	SD (µmol/l)	*N (n*)	Assay format		
					BrdU	MTS	CTG
Jurkat	Human acute T cell leukemia	0.0027	0.0022	4 (2)	1	2	1
MDA-MB-231	Human triple-negative breast cancer	0.0035	0.0050	4 (3)	4	—	—
KYSE-150	Human esophageal squamous cell carcinoma	0.0053	0.0040	8 (3)	5	3	—
MM.1s	Human multiple myeloma	0.0090	—	1 (1)	—	1	—
HL-60	Human acute myeloid leukemia	0.0130	—	1 (1)	—	1	—
HT-29	Human colorectal adenocarcinoma	0.0235	0.0134	2 (2)	1	—	1
MCF-7	Human breast carcinoma	0.0370	—	1 (1)	—	1	—
Loucy	Human T-acute lymphoblastic leukemia	<0.01	—	1 (1)	—	1	—
IMR-90	(Normal) Human fetal lung fibroblast	>1.0	—	2 (1)	1	—	1

‘—’, single experiment with no SD calculated; BrdU, 5-bromo-2′-deoxyuridine thymidine incorporation assay where the number indicates how many of each assay was performed; CTG, CellTiter-Glo Luminescent Cell Viability Assay; IC_50_, relative half-maximal inhibitory concentration (µmol/l) shown as mean for *N* > 1; MTS, CellTiter 96 AQueous One Solution Cell Proliferation Assay; *N (n*), ‘*N*’ number of experiments and ‘*n*’ different TACH101 batches tested.

In a larger screening study, TACH101 antiproliferative activity was determined across a broad cancer cell line panel composed of 301 cancer cell lines originating from 24 different hematologic and solid cancer types. Results demonstrate that TACH101 displayed a highly selective potency profile, with IC_50_ values ranging from less than 500 pM to more than 10 µmol/l. Among the 301 cell lines, 209 were inhibited by TACH101 with IC_50_ values < 1 µmol/l, whereas 89 lines were insensitive with IC_50_ values ≥ 10 µmol/l (Supplementary Table S2, Supplemental Digital Content 1, http://links.lww.com/ACD/A498). The three cell lines had IC_50_ values between 1 and 10 µmol/l and were considered moderately sensitive.

The antiproliferative activity of TACH101 was also tested in patient-derived cancer organoid models. The IC_50_ values for TACH101 inhibition of colony formation and viability were determined for colon cancer organoid models (SU60, T002C, SU62, SU34, SU103, T035C, and SU106), pancreatic cancer organoid models (PA0165F, PA0143F, T016P, and T028P), and a breast cancer organoid model (FS53). TACH101 treatment demonstrated submicromolar antiproliferative activity for SU60, T002C, and SU62 CRC models yielding IC_50_ values < 0.15 µmol/l (Supplementary Table S4, Supplemental Digital Content 1, http://links.lww.com/ACD/A498). TACH101 treatment also demonstrated potent antiproliferative activity in the PA0165F pancreatic carcinoma model, yielding IC_50_ values < 0.03 µmol/l. The SU34, SU103, T035C, and SU106 CRC models and the pancreatic carcinoma models PA0143F, T016P, and T028P were unresponsive to TACH101 in these colony formation and viability assays, with IC_50_ values > 10 µmol/l. An IC_50_ value of 13.6 µmol/l was determined in the FS53 human breast carcinoma model following TACH101 treatment.

### TACH101 induces apoptosis and cell cycle arrest and reduces tumor-initiating cell populations

The ability of TACH101 to induce apoptosis has been investigated in various human cancer cell lines (human esophageal squamous cell carcinoma KYSE-150, human triple-negative breast cancer MDA-MB-231, human colorectal adenocarcinoma HT-29, and human breast cancer HCC1937). Results show that TACH101 induced apoptosis in KYSE-150, MDA-MB-231, and HT-29 cells with EC_50_ values of 0.033, 0.132, and 0.092 µmol/l, respectively (Table 3).

**Table 3:** TACH101 half-maximal effective concentration for apoptosis

Cell line	Description	EC_50_ (µmol/l)
KYSE-150	Human esophageal squamous cell carcinoma	0.033
MDA-MB-231	Human triple-negative breast cancer	0.132
HT-29	Human colorectal adenocarcinoma	0.092
HCC1937	Human triple-negative breast cancer	>100

EC_50_, relative half-maximal effective concentration (µmol/l).

**Fig. 3:**
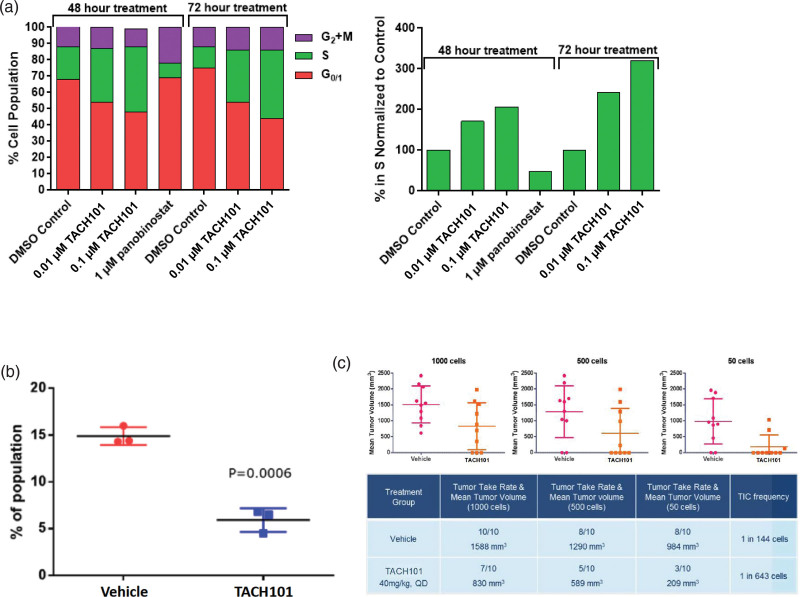
(a) TACH101 induces cell cycle arrest. Shown on left is the percent of MDA-MB-231 cell population in each cell cycle phase after treatment with DMSO negative control, 1 µmol/l Panobinostat positive control, or TACH101 at 0.01 and 0.1 µmol/l for 48 and 72 h. Shown on right is the number of cells in S as % of Control = (%Streated/%Scontrol) × 100. (b) Flow cytometric analysis of CD44^High^EpCAM^+^ cell population from SU60 xenograft tumors post TACH101 treatment. Flow cytometry analysis performed on single cell isolated from 3 vehicle-treated and 3 TACH101-treated tumors. *P*-value was determined with unpaired Student’s *t*-test. (c) Limiting Dilution Assay for SU60 xenograft tumors post TACH101 Treatment. Dissociated tumor cells (50–1000) from TACH101-treated and vehicle control groups were injected subcutaneously in a limiting dilution manner into NSG mice and tumor volumes were measured. NSG, nonobese diabetic/severe combined immunodeficiency gamma.

The effects of TACH101 on cell cycle progression were investigated in MDA-MB-231 cells. Treatment with TACH101 caused cells to arrest in S-phase: 33 and 40% of the population accumulated in S-phase in response to treatment with 0.01 and 0.1 µmol/l TACH101 for 24 h, respectively (Fig. 3a). Approximately 20% of DMSO-treated cells were in the S-phase. Longer TACH101 treatment resulted in more cells being arrested in the S-phase.

To determine the effects of KDM4 inhibition on tumor-initiating cell (TIC) frequency, we evaluated the tumorigenic potential of SU60 xenograft cells after treatment with TACH101 versus vehicle. TACH101 significantly reduced the proportion of tumorigenic cells (CD44^high^EpCAM^+^) by 2.5-fold compared with the vehicle control (Fig. 3b). In addition, limiting dilution assays showed that TACH101 reduced TIC frequency by 4.4-fold compared with vehicle-treated tumors (Fig. 3c).

### TACH101 induces potent tumor growth inhibition in xenograft models

**Fig. 4:**
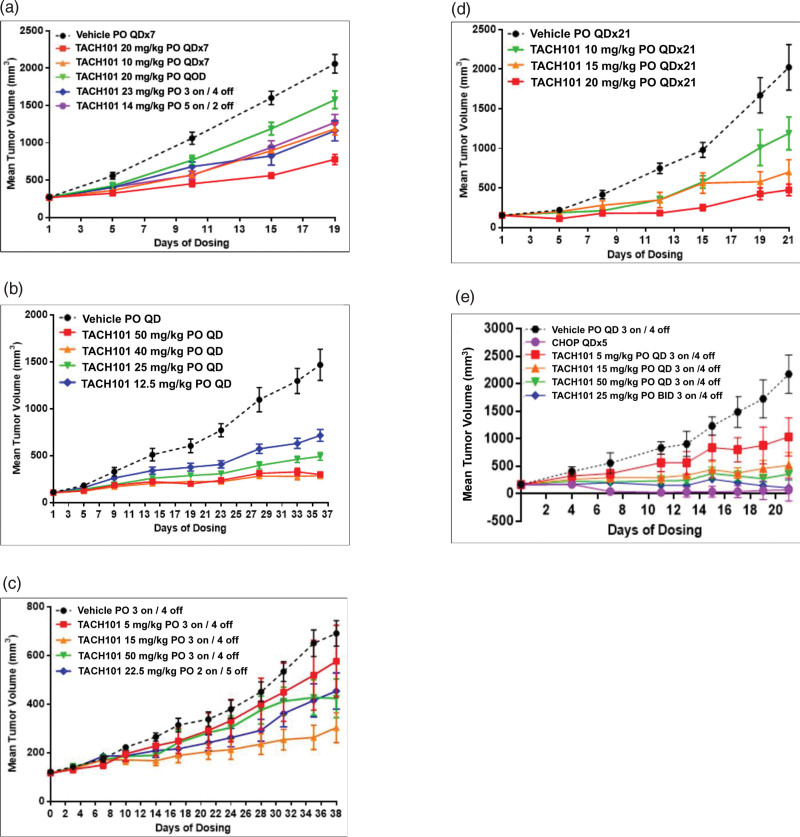
Efficacy of TACH101 on tumor growth inhibition in (a) SU60 colorectal cancer PDX model, (b) COH70 triple-negative breast cancer PDX model, (c) GXA-3036 gastric cancer PDX model, (d) KYSE-150 esophageal carcinoma CDX model, and (e) OCI-LY19 diffuse large B-cell lymphoma CDX model. CDX, cell line-derived xenograft; PDX, patient-derived xenograft.

Treatment with TACH101 resulted in potent dose-dependent tumor growth inhibition (TGI) in four patient-derived xenograft (PDX) models: SU60 CRC (Fig. 4a), COH70 TNBC (Fig. 4b), and GXA-3036 gastric cancer (Fig. 4c), and two human cell line-derived xenograft (CDX) models: KYSE-150 ESCC (Fig. 4d) and OCI-LY19 DLBCL (Fig. 4e). In the SU60 PDX model, TACH101 at 10 or 20 mg/kg administered with a schedule of QD ×7 per week yielded dose-dependent TGIs of 48 and 71%, respectively. Considering the same weekly total dose (70 mg/kg/week), the intermittent schedules of 3 on/4 off and 5 on/2 off resulted in similar tumor growth responses as QD ×7 (52 and 42% vs. 48% TGI). In the COH70 PDX model, TACH101 at 12.5, 25, 40, or 50 mg/kg administered QD ×36 yielded dose-dependent TGIs of 57, 68, 86, and 86%, respectively. TACH101 inhibition of tumor growth seemed to reach a plateau at doses ≥40 mg/kg. In the GXA-3036 PDX model, TACH101 at 5, 15, or 50 mg/kg administered on the 3 on/4 off schedule yielded TGIs of 43, 69, and 52%, respectively. The lower TGI exhibited by the 5 mg/kg group was due to a moderate increase in tumor volume from a single animal, which shifted the median. TACH101 at 22. 5 mg/kg administered on a 2 on/5 off schedule yielded a TGI of 41%. Differences in median net tumor volumes on the day of TGI analyses for treated versus control animals were significant for TACH101 at 15 mg/kg (*P* = 0.0007), but not for the other treatment regimens (*P* > 0.05). In the KYSE-150 CDX model, TACH101 at 10, 15, or 20 mg/kg administered QD x21 yielded dose-dependent TGIs of 54, 76, and 84%, respectively. In the OCI-LY19 CDX model, TACH101 at 5, 15, or 50 mg/kg administered QD on a 3 on/4 off schedule yielded dose-dependent TGIs of 55, 83, and 90%, respectively. TACH101 at 25 mg/kg administered BID on the 3 on/4 off yielded a TGI of 102% (with three of eight complete remissions), which was similar to that achieved with cyclophosphamide, doxorubicin, vincristine, and prednisone (CHOP) (108%; seven of eight complete remissions), the gold standard first-line chemotherapy regimen for DLBCL.

## Discussion

Epigenetic dysregulation is associated with oncogenesis and tumor proliferation in multiple cancers. Drugs targeting epigenetic enzymes such as inhibitors of histone deacetylase (HDAC), histone methyl transferases (HMTs such as EZH2), and lysine demethylase 1 (LSD1 or KDM1A) have entered clinical trials, some of which are in phase 3. Furthermore, four HDAC inhibitors (Vorinostat, Romidepsin, Panobinostat, and Belinostat), and one EZH2 (HMT) inhibitor (tazemetostat) have been approved by the United States Food and Drug Administration.

We identified TACH101 as a first-in-class small molecule with selective and potent pan-KDM4 inhibitory activities. In this study, we characterized the mechanisms of inhibition and selectivity in a series of histone demethylation assays. It should be emphasized that while TACH101 targets all four KDM4 isotypes (A–D), it has no activity toward other KDM members or enzymes. In addition, the ability of TACH101 to inhibit multiple isoforms of KDM4 suggests its broad-spectrum activity against multiple tumorigenic mechanisms in human patients, where cancer is often complex and heterogeneous.

Currently, there are no known clinical trials for KDM4 inhibitors. TACH101, the first KDM4 inhibitor to be tested in humans, offers several advantages over other histone-modifying agents. First, TACH101 has the potential to be used in both hematologic and solid tumors because of its broad therapeutic range and activity against all forms of KDM4. Second, unlike KDM1 (or LSD1) inhibitors, a close competitor to TACH101 as both are histone lysine demethylases, TACH101 has the potential to elicit a greater impact because KDM4 enzymes demethylate not only di- and monomethylated lysine residues (e.g. H3K9me1/2) but also trimethylated lysine residues (e.g. H3K9me3), which the former cannot do. In addition, KDM4 has been shown to interact with proteins/genes known to play a role in genomic stability and, thus, may have a more direct effect on tumorigenesis.

Interestingly, cancers exhibiting distinct KDM4 overexpression profiles seem to correlate with a specific type or subtype of tumor. For example, basal breast cancer cells significantly overexpress KDM4A and D. In contrast, infiltrating duct carcinoma tends to exhibit high KDM4A levels, but fibroadenoma does not [44]. KDM4B overexpression is reported to be predominant in both estrogen receptor–positive breast cancer [45] and triple-negative breast cancer [46]. In lung cancer, KDM4C expression is higher in adenocarcinoma than in squamous cell carcinoma [47]. As a pan-KDM4 inhibitor, TACH101 is believed to be potent and efficacious against a broad range of cancers and associated tumor subtypes, as suggested by the results of in-vitro and in-vivo cancer cell lines and PDX models.

In summary, TACH101 is a novel, first-in-class, small-molecule inhibitor of the epigenetic gene regulatory target, KDM4. KDM4 is a family of histone lysine demethylases that, when overexpressed or deregulated, drives key processes linked to cancer hallmarks such as cell proliferation, evasion of apoptosis, metastasis, deficiency in DNA repair, and genomic instability. KDM4 also plays an important role in epigenetic regulation of embryonic stem cell identity and self-renewal of cancer stem cells. Inhibition of KDM4 activity is believed to have profound anticancer effects by affecting the transcriptome and other nuclear processes during the initiation and progression of cancer. TACH101 represents a new therapeutic approach for cancers refractory to conventional therapies. A phase 1 clinical trial of TACH101 in patients with advanced and metastatic tumors has been initiated and is currently ongoing (NCT05076552).

## Acknowledgements

The authors would like to thank the following former employees of Celgene Quanticel Research for their skillful work and valuable scientific insight: Vivek Chopra, Antonia Lopez-Girona, Aaron Nguyen, Nisha Patel, Ryan Stansfield, Calvin Vu, and Natalie Yuen. The authors would also like to thank Dr. Stephen Quake for providing his expertise and helpful discussions.

All works were sponsored by Celgene Quanticel Research and Tachyon Therapeutics, Inc.

### Conflicts of interest

C.C., V.D., and F.P. are employees of Tachyon Therapeutics, Inc.; F.P., J.S., and M.C. are shareholders in Tachyon Therapeutics, Inc. For the remaining authors, there are no conflicts of interest.

## Supplementary Material

## References

[1] Cloos PA Christensen J Agger K Maiolica A Rappsilber J Antal T. The putative oncogene GASC1 demethylates tri- and dimethylated lysine 9 on histone H3. Nature 2006; 442:307–311. 16732293 10.1038/nature04837

[2] Klose RJ Yamane K Bae Y Zhang D Erdjument-Bromage H Tempst P. The transcriptional repressor JHDM3A demethylates trimethyl histone H3 lysine 9 and lysine 36. Nature 2006; 442:312–316. 16732292 10.1038/nature04853

[3] Fodor BD Kubicek S Yonezawa M O’Sullivan RJ Sengupta R Perez-Burgos L. Jmjd2b antagonizes H3K9 trimethylation at pericentric heterochromatin in mammalian cells. Genes Dev 2006; 20:1557–1562. 16738407 10.1101/gad.388206 PMC1482475

[4] Young LC Hendzel MJ. The oncogenic potential of Jumonji D2 (JMJD2/KDM4) histone demethylase overexpression. Biochem Cell Biol 2013; 91:369–377. 24219278 10.1139/bcb-2012-0054

[5] Cascante A Klum S Biswas M Antolin-Fontes B Barnabe-Heider F Hermanson O. Gene-specific methylation control of H3K9 and H3K36 on neurotrophic BDNF versus astroglial GFAP genes by KDM4A/C regulates neural stem cell differentiation. J Mol Biol 2014; 426:3467–3477. 24747049 10.1016/j.jmb.2014.04.008

[6] Trojer P Zhang J Yonezawa M Schmidt A Zheng H Jenuwein T. Dynamic histone H1 isotype 4 methylation and demethylation by histone lysine methyltransferase G9a/KMT1C and the Jumonji Domain-containing JMJD2/KDM4 proteins. J Biol Chem 2009; 284:8395–8405. 19144645 10.1074/jbc.M807818200 PMC2659197

[7] Pedersen MT Kooistra SM Radzisheuskaya A Laugesen A Johansen JV Hayward DG. Continual removal of H3K9 promoter methylation by Jmjd2 demethylases is vital for ESC self-renewal and early development. EMBO J 2016; 35:1550–1564. 27266524 10.15252/embj.201593317 PMC4899663

[8] Agger K Miyagi S Pedersen MT Kooistra SM Johansen JV Helin K. Jmjd2/Kdm4 demethylases are required for expression of Il3ra and survival of acute myeloid leukemia cells. Genes Dev 2016; 30:1278–1288. 27257215 10.1101/gad.280495.116 PMC4911927

[9] Ding X Pan H Li J Zhong Q Chen X Dry SM. Epigenetic activation of AP1 promotes squamous cell carcinoma metastasis. Sci Signal. 2013; 6:ra28, 1–13, S0–5. 10.1126/scisignal.2003884 PMC3951265 23633675

[10] Wissmann M Yin N Muller JM Greschik H Fodor BD Jenuwein T. Cooperative demethylation by JMJD2C and LSD1 promotes androgen receptor-dependent gene expression. Nat Cell Biol 2007; 9:347–353. 17277772 10.1038/ncb1546

[11] Yang ZQ Imoto I Fukuda Y Pimkhaokham A Shimada Y Imamura M. Identification of a novel gene, GASC1, within an amplicon at 9p23-24 frequently detected in esophageal cancer cell lines. Cancer Res 2000; 60:4735–4739. 10987278

[12] Berry WL Shin S Lightfoot SA Janknecht R. Oncogenic features of the JMJD2A histone demethylase in breast cancer. Int J Oncol 2012; 41:1701–1706. 22948256 10.3892/ijo.2012.1618

[13] Liu G Bollig-Fischer A Kreike B van de Vijver MJ Abrams J Ethier SP. Genomic amplification and oncogenic properties of the GASC1 histone demethylase gene in breast cancer. Oncogene 2009; 28:4491–4500. 19784073 10.1038/onc.2009.297 PMC2795798

[14] Shi L Sun L Li Q Liang J Yu W Yi X. Histone demethylase JMJD2B coordinates H3K4/H3K9 methylation and promotes hormonally responsive breast carcinogenesis. Proc Natl Acad Sci U S A 2011; 108:7541–7546. 21502505 10.1073/pnas.1017374108 PMC3088624

[15] Yamamoto S Tateishi K Kudo Y Yamamoto K Isagawa T Nagae G. Histone demethylase KDM4C regulates sphere formation by mediating the cross talk between Wnt and Notch pathways in colonic cancer cells. Carcinogenesis 2013; 34:2380–2388. 23698634 10.1093/carcin/bgt174

[16] Kogure M Takawa M Cho HS Toyokawa G Hayashi K Tsunoda T. Deregulation of the histone demethylase JMJD2A is involved in human carcinogenesis through regulation of the G(1)/S transition. Cancer Lett 2013; 336:76–84. 23603248 10.1016/j.canlet.2013.04.009

[17] Shin S Janknecht R. Activation of androgen receptor by histone demethylases JMJD2A and JMJD2D. Biochem Biophys Res Commun 2007; 359:742–746. 17555712 10.1016/j.bbrc.2007.05.179

[18] Rui L Emre NC Kruhlak MJ Chung HJ Steidl C Slack G. Cooperative epigenetic modulation by cancer amplicon genes. Cancer Cell 2010; 18:590–605. 21156283 10.1016/j.ccr.2010.11.013 PMC3049192

[19] Luo W Chang R Zhong J Pandey A Semenza GL. Histone demethylase JMJD2C is a coactivator for hypoxia-inducible factor 1 that is required for breast cancer progression. Proc Natl Acad Sci U S A 2012; 109:E3367–E3376. 23129632 10.1073/pnas.1217394109 PMC3523832

[20] Metzger E Stepputtis SS Strietz J Preca BT Urban S Willmann D. KDM4 inhibition targets breast cancer stem-like cells. Cancer Res 2017; 77:5900–5912. 28883001 10.1158/0008-5472.CAN-17-1754

[21] Wang W Oguz G Lee PL Bao Y Wang P Terp MG. KDM4B-regulated unfolded protein response as a therapeutic vulnerability in PTEN-deficient breast cancer. J Exp Med 2018; 215:2833–2849. 30266800 10.1084/jem.20180439 PMC6219741

[22] Ma D Liu H Qin Y Tian Z Li S Liang N. KLF8 overexpression promotes the growth of human lung cancer cells by promoting the expression of JMJD2A. Cancer Cell Int 2019; 19:258. 31624471 10.1186/s12935-019-0970-3 PMC6781403

[23] Chen L Fu L Kong X Xu J Wang Z Ma X. Jumonji domain-containing protein 2B silencing induces DNA damage response via STAT3 pathway in colorectal cancer. Br J Cancer 2014; 110:1014–1026. 24473398 10.1038/bjc.2013.808 PMC3929886

[24] Deng WW Hu Q Liu ZR Chen QH Wang WX Zhang HG. KDM4B promotes DNA damage response via STAT3 signaling and is a target of CREB in colorectal cancer cells. Mol Cell Biochem 2018; 449:81–90. 29633065 10.1007/s11010-018-3345-5

[25] Fu LN Wang YQ Tan J Xu J Gao QY Chen YX. Role of JMJD2B in colon cancer cell survival under glucose-deprived conditions and the underlying mechanisms. Oncogene 2018; 37:389–402. 28945223 10.1038/onc.2017.345

[26] Kim H Abd Elmageed ZY Davis C El-Bahrawy AH Naura AS Ekaidi I. Correlation between PDZK1, Cdc37, Akt and breast cancer malignancy: the role of PDZK1 in cell growth through Akt stabilization by increasing and interacting with Cdc37. Mol Med 2014; 20:270–279. 24869908 10.2119/molmed.2013.00166 PMC4107102

[27] Kim JG Yi JM Park SJ Kim JS Son TG Yang K. Histone demethylase JMJD2B-mediated cell proliferation regulated by hypoxia and radiation in gastric cancer cell. Biochim Biophys Acta 2012; 1819:1200–1207. 23046878 10.1016/j.bbagrm.2012.10.001

[28] Li H Yang X Wang G Li X Tao D Hu J. KDM4B plays an important role in mitochondrial apoptosis by upregulating HAX1 expression in colorectal cancer. Oncotarget 2016; 7:57866–57877. 27506941 10.18632/oncotarget.11077 PMC5295396

[29] Li W Zhao L Zang W Liu Z Chen L Liu T. Histone demethylase JMJD2B is required for tumor cell proliferation and survival and is overexpressed in gastric cancer. Biochem Biophys Res Commun 2011; 416:372–378. 22133676 10.1016/j.bbrc.2011.11.045

[30] Sun BB Fu LN Wang YQ Gao QY Xu J Cao ZJ. Silencing of JMJD2B induces cell apoptosis via mitochondria-mediated and death receptor-mediated pathway activation in colorectal cancer. J Dig Dis 2014; 15:491–500. 24957706 10.1111/1751-2980.12166

[31] Wu X Li R Song Q Zhang C Jia R Han Z. JMJD2C promotes colorectal cancer metastasis via regulating histone methylation of MALAT1 promoter and enhancing beta-catenin signaling pathway. J Exp Clin Cancer Res 2019; 38:435. 31665047 10.1186/s13046-019-1439-x PMC6819649

[32] Zheng H Chen L Pledger WJ Fang J Chen J. p53 promotes repair of heterochromatin DNA by regulating JMJD2b and SUV39H1 expression. Oncogene 2014; 33:734–744. 23376847 10.1038/onc.2013.6 PMC3912226

[33] Hu CE Liu YC Zhang HD Huang GJ. JMJD2A predicts prognosis and regulates cell growth in human gastric cancer. Biochem Biophys Res Commun 2014; 449:1–7. 24802408 10.1016/j.bbrc.2014.04.126

[34] Zhao L Li W Zang W Liu Z Xu X Yu H. JMJD2B promotes epithelial-mesenchymal transition by cooperating with beta-catenin and enhances gastric cancer metastasis. Clin Cancer Res 2013; 19:6419–6429. 24077348 10.1158/1078-0432.CCR-13-0254

[35] Kim TD Jin F Shin S Oh S Lightfoot SA Grande JP. Histone demethylase JMJD2A drives prostate tumorigenesis through transcription factor ETV1. J Clin Invest 2016; 126:706–720. 26731476 10.1172/JCI78132 PMC4731184

[36] Lin CY Wang BJ Chen BC Tseng JC Jiang SS Tsai KK. Histone demethylase KDM4C stimulates the proliferation of prostate cancer cells via activation of AKT and c-Myc. Cancers (Basel) 2019; 11:1785. 31766290 10.3390/cancers11111785 PMC6896035

[37] Sha J Han Q Chi C Zhu Y Pan J Dong B. Upregulated KDM4B promotes prostate cancer cell proliferation by activating autophagy. J Cell Physiol 2020; 235:2129–2138. 31468537 10.1002/jcp.29117

[38] Wilson C Qiu L Hong Y Karnik T Tadros G Mau B. The histone demethylase KDM4B regulates peritoneal seeding of ovarian cancer. Oncogene 2017; 36:2565–2576. 27869162 10.1038/onc.2016.412 PMC5418103

[39] Das PP Shao Z Beyaz S Apostolou E Pinello L De Los Angeles A. Distinct and combinatorial functions of Jmjd2b/Kdm4b and Jmjd2c/Kdm4c in mouse embryonic stem cell identity. Mol Cell 2014; 53:32–48. 24361252 10.1016/j.molcel.2013.11.011 PMC3919500

[40] Wang J Zhang M Zhang Y Kou Z Han Z Chen DY. The histone demethylase JMJD2C is stage-specifically expressed in preimplantation mouse embryos and is required for embryonic development. Biol Reprod 2010; 82:105–111. 19696013 10.1095/biolreprod.109.078055

[41] Loh YH Zhang W Chen X George J Ng HH. Jmjd1a and Jmjd2c histone H3 Lys 9 demethylases regulate self-renewal in embryonic stem cells. Genes Dev 2007; 21:2545–2557. 17938240 10.1101/gad.1588207 PMC2000320

[42] Kim J Woo AJ Chu J Snow JW Fujiwara Y Kim CG. A Myc network accounts for similarities between embryonic stem and cancer cell transcription programs. Cell 2010; 143:313–324. 20946988 10.1016/j.cell.2010.09.010 PMC3018841

[43] Liao TT Lin CC Jiang JK Yang SH Teng HW Yang MH. Harnessing stemness and PD-L1 expression by AT-rich interaction domain-containing protein 3B in colorectal cancer. Theranostics 2020; 10:6095–6112. 32483441 10.7150/thno.44147 PMC7255042

[44] Li BX Li J Luo CL Zhang MC Li H Li LL. Expression of JMJD2A in infiltrating duct carcinoma was markedly higher than fibroadenoma, and associated with expression of ARHI, p53 and ER in infiltrating duct carcinoma. Indian J Exp Biol 2013; 51:208–217. 23678541

[45] Ye Q Holowatyj A Wu J Liu H Zhang L Suzuki T. Genetic alterations of KDM4 subfamily and therapeutic effect of novel demethylase inhibitor in breast cancer. Am J Cancer Res 2015; 5:1519–1530. 26101715 PMC4473328

[46] Slee RB Steiner CM Herbert BS Vance GH Hickey RJ Schwarz T. Cancer-associated alteration of pericentromeric heterochromatin may contribute to chromosome instability. Oncogene 2012; 31:3244–3253. 22081068 10.1038/onc.2011.502

[47] Uimonen K Merikallio H Paakko P Harju T Mannermaa A Palvimo J. GASC1 expression in lung carcinoma is associated with smoking and prognosis of squamous cell carcinoma. Histol Histopathol 2014; 29:797–804. 24371038 10.14670/HH-29.797

