# Supplementary material for: TACH101, a first-in-class pan-inhibitor of KDM4 histone demethylase
Source: Anticancer Drugs. 2023 Mar 24;34(10):1122–31. doi: 10.1097/CAD.0000000000001514 (PMC10569680; doi:10.1097/CAD.0000000000001514)
Supplement: Supplementary file 1 [file acd-34-1122-s001.pdf]

SUPPLEMENTAL METHODS

Mouse xenograft studies

For the human colorectal cancer model, 2 × 10^6 SU60 cells (0.1 ml) were suspended in Matrigel and inoculated subcutaneously in female NSG mice. When the average tumor size reached ~270 mm^3, animals were randomized into 6 groups (8 mice per group) and treatments were initiated. Vehicle control mice received 0.1 ml of the vehicle consisting of 50% polyethylene glycol 400 (PEG 400) and 50% PBS (pH 9) for 7 consecutive days per week until end of experiment (Day 19). Treated mice received TACH101 in vehicle at 102 or 20 mg/kg QD x7, at 20 mg/kg QOD, at 23 mg/kg 3 on/4 off, or at 14 mg/kg at 5 on/2 off, until Day 19 when tumor growth inhibition (TGI) was determined as (1 - $Diff_{treated}/Diff_{control}$) x 100.

For the human esophageal cancer model, 1 × 10^7 KYSE-150 cells (0.1 ml) were suspended in Matrigel and inoculated subcutaneously in female NSG mice. When the average tumor size reached ~153 mm^3, animals were randomized into 4 groups consisting of 8 mice per group and treatments were initiated. Control mice received 10% PEG 400 and 90% of 0.5% methylcellulose (MC) vehicle PO QD x21. Treated mice received TACH101 in vehicle at 10, 15, or 20 mg/kg PO on the same schedule. TGI was determined on Day 21 as (1 - $Diff_{treated}/Diff_{control}$) x 100.

For the human triple negative breast cancer model, 2 × 10^6 COH70 cells (0.2 ml) were suspended in Matrigel and inoculated subcutaneously in female NSG mice. When the average tumor size reached ~107 mm^3, animals were randomized into 5 groups consisting of 9 mice per group and treatments were initiated. Control mice received PBS (pH 10) vehicle PO QD x36. Treated mice received TACH101 (dissolved in PBS + 2 equivalents of NaOH, final pH 10) PO at 12.5, 25, 40,

or 50 mg/kg on a QD x36 schedule. TGI was determined on Day 36 as (1 - $Diff_{treated}/Diff_{control}$) x 100.

For the human gastric adenocarcinoma cancer model, GXA-3036 tumor fragment (3 to 4 mm edge length) were inoculated subcutaneously in female immunodeficient NMRI-$Foxn1^{nu}$ mice. When the average tumor size reached ~109 mm^3, animals were randomized into 5 groups consisting of 8 mice per group and treatments were initiated. Control mice received 5% HPBCD in 50 mM phosphate buffer (pH 7.4) vehicle PO 3 on / 4 off. Treated mice received TACH101 in vehicle at 5, 15, or 50 mg/kg on the same schedule or at 22.5 mg/kg 2 on / 5 off. TGI was determined on Day 38 as (1 - $Diff_{treated}/Diff_{control}$) x 100.

For the human Diffuse Large B-cell Non-Hodgkin's Lymphoma (DLBCL) model, 5×10^6 OCI-LY19 tumor cells (0.1 ml) suspended in Matrigel were inoculated subcutaneously in female SCID mice. When the average tumor size reached ~165 mm^3, animals were randomized into 6 groups consisting of 8 mice per group and treatments were initiated. Control mice received 0.5% MC vehicle PO QD 3 on/4 off and positive control mice received CHOP (cyclophosphamide, doxorubicin, vincristine, and prednisone) therapy QD x5. Treated mice received TACH101 in vehicle at 5, 15, or 50 mg/kg QD or at 25 mg/kg BID on the 3 on/4 off schedule. TGI was determined on Day 21 as (1 - $Diff_{treated}/Diff_{control}$) x 100.

For all models, individual tumors were measured twice weekly in two dimensions using a caliper, and the tumor volumes (TV) in mm^3 were calculated using either the formula: TV = 0.5 a x b^2, where a and b are the long and short tumor diameters in mm, respectively, or the formula: TV = 0.5 ($l \times w \times h$), where l, w, and h are the tumor length, width, and height in mm, respectively. Mean tumor volumes ± standard error of the mean (SEM) were calculated and plotted versus days of dosing. Body weights were measured twice weekly. Body weights ± SEM

were calculated and plotted versus days of dosing. The difference in mean net tumor volumes on the day of TGI analysis for treated versus control animals was evaluated statistically using one-way ANOVA followed by Dunnett's multiple comparisons test, or for the GXA-3036 model, using non-parametric Kruskal-Wallis test followed by Dunn's multiple comparisons test. Adjusted p-values are shown where a calculated probability $(p) \leq 0.05$ was considered statistically significant.

SUPPLEMENTAL TABLES

Table S1.

Initiate assay with KDM						
KDM Family Isoform	Initiate Rxn	Key Components			Detection	
	KDM (nM)	H3 biotinylated peptide (nM)	α-KG (μM)	Rxn time (min)	Anti-H3 Ab	Technology
KDM2A	0.15	H3K36me2 30	0.20	30	H3K36me1	AlphaScreen®
KDM2B	0.30	H3K36me2 30	0.20	30	H3K36me1	AlphaScreen®
KDM3A	1.00	H3K9me1 200	0.50	15	H3K9/K27	Lance® Detection
KDM4A	4.00	H3K9me3 300	2.00	35	H3K9me2	Lance® Detection
KDM4C	0.30 – 2.00	H3K9me3 300	2.00	35	H3K9me2	Lance® Detection
KDM5A	0.60 – 1.00	H3K4me3 300	2.00	35	H3K4me1-2	Lance® Detection
KDM5B	0.30 – 1.50	H3K4me3 300	2.00	35	H3K4me1-2	Lance® Detection
KDM5C	0.20	H3K4me3 300	2.00	35	H3K4me1-2	Lance® Detection
KDM6A	2.50	H3K27me3 250	1.00	20	H3K27me1	AlphaScreen®
KDM6B	1.00	H3K27me3 250	1.00	20	H3K27me1	AlphaScreen®
KDM7B	4.00	H3K9me1 200	0.50	15	H3K9/K27	Lance® Detection
**Pre-incubate KDM + TACH101 for 1 hour then initiate assay with α-KG and peptide**						
**KDM Family Isoform**	**Initiate Rxn**	**Key Components**			**Detection**	
	**α-KG (nM)**	**H3 biotinylated peptide (nM)**	**α-KG (μM)**	**Rxn time (min)**	**Anti-H3 Ab**	**Technology**
KDM2A	20	H3K36me2 30	0.15	20	H3K36me1	AlphaScreen®
KDM2B	20	H3K36me2 30	0.30	20	H3K36me1	AlphaScreen®
KDM4A	100	H3K9me3 300	4.00	20	H3K9me2	Lance® Detection
KDM4B	100	H3K9me3 300	0.30	20	H3K9me2	Lance® Detection
KDM4C	100	H3K9me3 300	0.30 – 2.00	20	H3K9me2	Lance® Detection
KDM4D	100	H3K9me3 300	4.00	20	H3K9me2	Lance® Detection

KDM4E	100	H3K9me3 300	4.00	20	H3K9me2	Lance® Detection
KDM5A	100	H3K4me3 300	0.60 – 1.00	20	H3K9me1-2	Lance® Detection
KDM5B	100	H3K4me3 300	0.30 – 1.50	20	H3K9me1-2	Lance® Detection
KDM6A	50	H3K27me3 250	1.00	20	H3K27me1	AlphaScreen®
KDM6B	50	H3K27me3 250	1.00	20	H3K27me1	AlphaScreen®

KDM selectivity panel enzymatic assay methods and conditions. Rxn = reaction; nM = nanomolar; μM = micromolar; α-KG = alpha-ketoglutarate; min = minutes; Ab = antibody

Table S2.

**Cell line**	**IC_{50} (μM)**	**Tumor Type**
NCIH1618	5.00E-04	lung
NCIH1048	0.000706	lung
NCIH1155	0.000765	lung
NCIH1666	0.001165	lung
REH	0.001210	hematopoietic and lymphoid tissue
MOLT4	0.001250	hematopoietic and lymphoid tissue
NCIH209	0.001307	lung
JURKAT	0.001488	hematopoietic and lymphoid tissue
NCIH1694	0.001753	lung
HCT15	0.001791	large intestine
JEKO1	0.001804	hematopoietic and lymphoid tissue
TALL1	0.001805	hematopoietic and lymphoid tissue
A2780	0.002137	ovary
KYSE30	0.002426	esophagus
LOUCY	0.002474	hematopoietic and lymphoid tissue
DLD1	0.002533	large intestine
U937	0.002554	hematopoietic and lymphoid tissue
NCIH2081	0.002568	lung
TF1	0.002586	hematopoietic and lymphoid tissue
RL	0.002993	hematopoietic and lymphoid tissue
NCIH2023	0.003031	lung
NAMALWA	0.003206	hematopoietic and lymphoid tissue
TE14	0.003393	esophagus
KYSE510	0.003395	esophagus
DMS79	0.003467	lung
NCIH2286	0.003508	lung
NCIH82	0.003543	lung
NCIH2087	0.003555	lung

**Cell line**	**IC_{50} (μM)**	**Tumor Type**
PF382	0.003685	hematopoietic and lymphoid tissue
YD38	0.003771	upper aerodigestive tract
HPAC	0.003928	pancreas
PECAPJ34CLONEC12	0.003966	upper aerodigestive tract
MV411	0.004017	hematopoietic and lymphoid tissue
AZ521	0.004189	small intestine
SNU16	0.004217	stomach
TE11	0.004280	esophagus
NCIN87	0.004292	stomach
P31FUJ	0.004324	hematopoietic and lymphoid tissue
CORL279	0.004345	lung
HUTU80	0.004361	small intestine
HCC95	0.004400	lung
CAKI1	0.004429	kidney
RT11284	0.004435	urinary tract
G401	0.004449	soft tissue
K562	0.004556	hematopoietic and lymphoid tissue
MC116	0.004605	hematopoietic and lymphoid tissue
COLO320HSR	0.004609	large intestine
LNCAP	0.004648	prostate
NOMO1	0.005012	hematopoietic and lymphoid tissue
KYSE150	0.005097	esophagus
KYSE270	0.005351	esophagus
C32	0.005360	skin
SKMEL28	0.005562	skin
HCC1599	0.005645	breast
WSUDLCL2	0.005671	hematopoietic and lymphoid tissue
MKN1	0.005748	stomach
NCIH520	0.005895	lung

Cell line	IC_{50} (μM)	Tumor Type
NCIH1836	0.006075	lung
CHL1	0.006077	skin
5637	0.006107	urinary tract
THP1	0.006150	hematopoietic and lymphoid tissue
AGS	0.006186	stomach
TOV112D	0.006227	ovary
CAOV3	0.006468	ovary
HCC1187	0.006507	breast
KARPAS422	0.006525	hematopoietic and lymphoid tissue
NCIH358	0.006571	lung
TE6	0.006677	esophagus
T.T	0.006752	esophagus
NCIH524	0.006853	lung
TE9	0.006890	esophagus
HSC4	0.006951	upper aerodigestive tract
COLO680N	0.006972	esophagus
MIAPACA2	0.007021	pancreas
NCIH69	0.007089	lung
SKBR3	0.007137	breast
NCIH1092	0.007160	lung
KM12	0.007345	large intestine
DV90	0.007364	lung
FTC133	0.007491	thyroid
NCIH1299	0.007498	lung
NUGC3	0.007720	stomach
OCUM1	0.007790	stomach
HSC2	0.007994	upper aerodigestive tract
NCIH1703	0.008047	lung
KYSE410	0.008253	esophagus

**Cell line**	**IC_{50} (μM)**	**Tumor Type**
NCIH2291	0.008405	lung
TE5	0.008472	esophagus
NCIH1436	0.008754	lung
U2OS	0.009007	bone
SKMEL1	0.009242	skin
SW1463	0.009244	large intestine
RD	0.009294	soft tissue
HL60	0.009317	hematopoietic and lymphoid tissue
NCIH716	0.009514	large intestine
NCIH661	0.009732	lung
SW480	0.009788	large intestine
NCIH647	0.009841	lung
BFTC905	0.009914	urinary tract
BT549	0.009983	breast
NCIH508	0.010235	large intestine
KU1919	0.010268	urinary tract
COLO741	0.010360	skin
MDAMB453	0.010402	breast
SCC4	0.010532	upper aerodigestive tract
LU65	0.010583	lung
NUGC4	0.010595	stomach
A375	0.010596	skin
HCC70	0.010990	breast
A101D	0.011003	skin
HMCB	0.011093	skin
HLE	0.011279	liver
COLO205	0.011489	large intestine
RKO	0.011534	large intestine
NCIH460	0.011582	lung

Cell line	IC_{50} (μM)	Tumor Type
SNB19	0.011607	central nervous system
HEL	0.011746	hematopoietic and lymphoid tissue
HUH1	0.011768	liver
KMS11	0.012224	hematopoietic and lymphoid tissue
647V	0.012240	urinary tract
RPMI8226	0.012490	hematopoietic and lymphoid tissue
TE4	0.012665	esophagus
SW780	0.013009	urinary tract
HCC1806	0.013019	breast
NCIH2347	0.013060	lung
NCIH1437	0.013132	lung
SHSY5Y	0.013453	autonomic ganglia
MKN45	0.013529	stomach
HS578T	0.013853	breast
HUH7	0.013955	liver
HT1080	0.014147	soft tissue
HT55	0.014167	large intestine
MESSA	0.014307	soft tissue
143B	0.014366	bone
PC3	0.014471	prostate
U87MG	0.014695	central nervous system
CALU6	0.014859	lung
DAUDI	0.014888	hematopoietic and lymphoid tissue
KNS62	0.014901	lung
SCABER	0.015202	urinary tract
CFPAC1	0.015268	pancreas
NCIH1793	0.015288	lung
TE10	0.016273	esophagus
NCIH1944	0.016307	lung

Cell line	IC_{50} (μM)	Tumor Type
LS411N	0.016662	large intestine
DETROIT562	0.016685	upper aerodigestive tract
SKMEL5	0.016833	skin
NCIH522	0.017390	lung
GRANTA519	0.017643	hematopoietic and lymphoid tissue
DU145	0.017720	prostate
SNU398	0.017992	liver
HLF	0.018027	liver
NCIH1623	0.018862	lung
SNU668	0.018880	stomach
BXPC3	0.019388	pancreas
NCIH1930	0.020006	lung
HCT116	0.020431	large intestine
NCIH1355	0.020573	lung
T84	0.020979	large intestine
DMS273	0.022219	lung
ECGI10	0.022440	esophagus
786O	0.022468	kidney
HCC78	0.023920	lung
NCIH2122	0.023938	lung
OVISE	0.023953	ovary
LUDLU1	0.025828	lung
GP2D	0.026231	large intestine
WM2664	0.027533	skin
PLCPRF5	0.027960	liver
SW620	0.029260	large intestine
SKCO1	0.030585	large intestine
A549	0.032375	lung
NCIH929	0.034475	hematopoietic and lymphoid tissue

**Cell line**	**IC_{50} (μM)**	**Tumor Type**
ACHN	0.034708	kidney
NCIH1975	0.034766	lung
PANC0213	0.035795	pancreas
HT1376	0.036268	urinary tract
HT29	0.037031	large intestine
UMUC3	0.039504	urinary tract
SNU761	0.039689	liver
SKLU1	0.040003	lung
HCC1954	0.040563	breast
NCIH23	0.042711	lung
KATOIII	0.043772	stomach
A2058	0.046764	skin
SKMES1	0.047461	lung
SW48	0.050006	large intestine
HEPG2	0.052079	liver
SNUC2A	0.052512	large intestine
LOVO	0.057826	large intestine
HSC3	0.060326	upper aerodigestive tract
JIMT1	0.061923	breast
SW1710	0.063291	urinary tract
RERFLCMS	0.065898	lung
HS294T	0.066791	skin
ASPC1	0.066825	pancreas
PANC1	0.068580	pancreas
CAPAN1	0.115609	pancreas
NCIH446	0.119463	lung
HEP3B217	0.129298	liver
NCIH2172	0.132833	lung
SKMEL2	0.139680	skin

**Cell line**	**IC$_{50}$ (µM)**	**Tumor Type**
SNU5	0.146396	stomach
NCIH2228	0.178529	lung
BICR18	0.194270	upper aerodigestive tract
GI1	0.287440	central nervous system
MCF7	0.434714	breast
SKMEL3	0.472006	skin
WM115	0.477738	skin
COLO679	2.044655	skin
SUPT1	3.333300	hematopoietic and lymphoid tissue
SNGM	4.901703	endometrium
NCIH2171	10	lung
KNS81	10	central nervous system
MPP89	10	pleura
SNU886	10	liver
COLO829	10	skin
KALS1	10	central nervous system
SNU878	10	liver
GAK	10	Unknown
OSRC2	10	kidney
RCM1	10	large intestine
NCIH1734	10	lung
DMS153	10	lung
J82	10	urinary tract
BICR22	10	upper aerodigestive tract
SW1990	10	pancreas
HCC1143	10	breast
HCC2935	10	lung
KYSE70	10	esophagus
MDAMB468	10	breast

**Cell line**	**IC$_{50}$ (μM)**	**Tumor Type**
NMCG1	10	central nervous system
SKHEP1	10	liver
SW837	10	large intestine
A204	10	soft tissue
HCC1419	10	breast
BT474	10	breast
MEWO	10	skin
MDAMB231	10	breast
CALU3	10	lung
SKNSH	10	autonomic ganglia
ONS76	10	central nervous system
MDAMB175VII	10	breast
SKMEL31	10	skin
DMS53	10	lung
SNUC1	10	large intestine
NCIH1573	10	lung
NCIH1435	10	lung
NIHOVCAR3	10	ovary
SCC25	10	upper aerodigestive tract
NCIH441	10	lung
HCC1428	10	breast
VCAP	10	prostate
NCIH1838	10	lung
SKMEL24	10	skin
LU99	10	lung
NCIH2009	10	lung
RPMI7951	10	skin
NCIH596	10	lung
TE8	10	esophagus

Cell line	IC_{50} (µM)	Tumor Type
SW1271	10	lung
C2BBE1	10	large intestine
CAL27	10	upper aerodigestive tract
MDAMB157	10	breast
UACC812	10	breast
MDAMB436	10	breast
NCIH1395	10	lung
HS739T	10	breast
KURAMOCHI	10	ovary
NCIH1650	10	lung
CCFSTTG1	10	central nervous system
CAPAN2	10	pancreas
HCC827	10	lung
KASUMI1	10	hematopoietic and lymphoid tissue
HS852T	10	skin
HT144	10	skin
HCC1937	10	breast
BT20	10	breast
SW579	10	thyroid
HS695T	10	skin
T24	10	urinary tract
TE1	10	esophagus
MDAMB415	10	breast
SCC9	10	upper aerodigestive tract
SCC15	10	upper aerodigestive tract
KG1	10	hematopoietic and lymphoid tissue
SW1417	10	large intestine
NCIH1563	10	lung
DAOY	10	central nervous system

Cell line	IC_{50} (μM)	Tumor Type
HUH28	10	biliary tract
HCC38	10	breast
MALME3M	10	skin
SKOV3	10	ovary
G361	10	skin
SAOS2	10	bone
TE15	10	esophagus
HS839T	10	skin
HS688AT	10	skin
MDAMB361	10	breast
CALU1	10	lung
NCIH1755	10	lung

TACH101 potencies for 301 cell lines. IC_{50} = half-maximal inhibitory concentration (μM); μM = micromolar.

Table S3:

**TACH101 Half-Maximal Inhibitory Concentration (μM)**		
**Assay Conditions**	**Initiate assay with KDM**	**Pre-incubate KDM + TACH101 then initiate assay with α-KG**
**KDM4 Isoforms**		
KDM4C	0.66 ± 0.40	0.041 ± 0.013
KDM4D	---	0.050
KDM4B	---	0.058
KDM4A	0.36	0.080
KDM4E	---	0.270
**Other KDM Family Member Isoforms**		
KDM5B	0.14 ± 0.07	0.18 ± 0.11
KDM5C	0.24	---
KDM5A	0.40 ± 0.08	4.90
KDM6B	1.50 ± 0.9	> 1
KDM2A	3.40	> 20
KDM3A	4.90	---
KDM2B	6.40 ± 1.30	> 20
KDM6A	6.90	> 10
KDM7B	> 20	---

Selectivity and potency of TACH101 for members of the KDM family. Numbers that show ± SD are for independent experiments > 1.

Table S4:

**Description**	**Organoid Model**	**IC_{50} (µM)**
Human Colorectal Carcinoma	SU60	0.023
		0.064
	T002C	0.022
	SU62	0.149
	SU34	> 10
	SU103	> 10
	T035C	> 10
	SU106	> 10
Human Pancreatic Carcinoma	PA0165F	0.029
		0.016
	PA0143F	> 10
	T016P	> 10
	T028P	> 10
Human Breast Carcinoma	FS53	13.6

TACH101 half-maximal inhibition values for organoid cancer models. IC_{50} = half-maximal inhibitory concentration (µM); N = number of independent experiments.

SUPPLEMENTAL FIGURES

Figure S1:

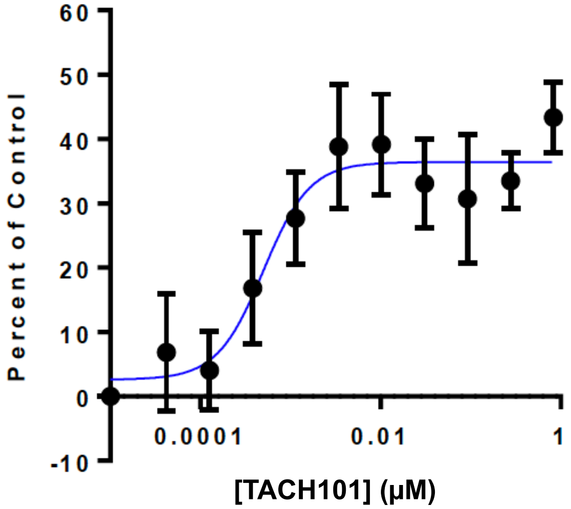

TACH101 inhibition of H3K36me3 demethylation in KYSE-150^{+KDM4C} cells in HTRF assay. Percent of Control = endogenous H3K36me3 in TACH101-treated cells normalized to H3K36me3 in QC5843 positive control-treated cells, expressed as a percent; the plot combined data from three HTRF experiments; error bars are ±SD for combined data. The assays were performed as described above.
